# A Novel Underwater Acoustic Target Identification Method Based on Spectral Characteristic Extraction via Modified Adaptive Chirp Mode Decomposition

**DOI:** 10.3390/e25040669

**Published:** 2023-04-16

**Authors:** Zipeng Li, Kunde Yang, Xingyue Zhou, Shunli Duan

**Affiliations:** 1 School of Marine Science and Technology, Northwestern Polytechnical University, Xi’an 710072, China; 2 Key Laboratory of Ocean Acoustics and Sensing, Northwestern Polytechnical University, Ministry of Industry and Information Technology, Xi’an 710072, China

**Keywords:** adaptive chirp mode decomposition, non-local means denoising, feature extraction, ship-radiated noise

## Abstract

As is well-known, ship-radiated noise (SN) signals, which contain a large number of ship operating characteristics and condition information, are widely used in ship recognition and classification. However, it is still a great challenge to extract weak operating characteristics from SN signals because of heavy noise and non-stationarity. Therefore, a new mono-component extraction method is proposed in this paper for taxonomic purposes. First, the non-local means algorithm (NLmeans) is proposed to denoise SN signals without destroying its time-frequency structure. Second, adaptive chirp mode decomposition (ACMD) is modified and applied on denoised signals to adaptively extract mono-component modes. Finally, sub-signals are selected based on spectral kurtosis (SK) and then analyzed for ship recognition and classification. A simulation experiment and two application cases are used to verify the effectiveness of the proposed method and the results show its outstanding performance.

## 1. Introduction

As ocean trade plays an increasingly important role worldwide, ship faults and marine terrorist attacks have been causing greater losses. To ensure unimpeded access and safety of international sea lanes, reliable technologies for ship classification and tracking are of great concern [1,2]. SN-signal-based analysis, as one of the most effective methods of ship detection and classification, is a potential solution in this area [3]. However, non-stationarity and heavy noise caused by the harsh ocean environment make it scarcely possible to extract shafting characteristics from raw SN signals. Specifically, inner-product-based methods, such as Fourier transformation (FT) and wavelet transform (WT) are unqualified for multiple feature matching because of fixed basis functions [4,5]. Although double-tree complex wavelet and multiwavelet are developed to solve the problem, they merely build more fixed basis functions to deal with different features. Therefore, adaptive feature extraction methods are expected.

Hilbert-Huang transform (HHT), proposed by Huang in 1998, is an effective method for nonlinear and non-stationary signal analysis [6]. Its core, empirical mode decomposition (EMD), is totally a self-organizing method and is based on the local scale characteristics of the signal itself [7]. It decomposes the multi-modulation signal into a few intrinsic mode functions (IMFS), and each IMF is considered as a mono-component. Once born, EMD is widely used in the fields of mechanical failure diagnosis, biomedical science, and underwater acoustic signal analysis [8,9,10,11,12,13]. However, the result of EMD is highly dependent on the local extremum searching algorithm. The end effects in interpolation and Hilbert transform (HT) also possibly lead to IMF mixing, reducing the effectiveness of feature extraction [14].

In spite of disadvantages and lack of strict mathematical derivation, EMD is still a powerful tool for signal denoising and feature extraction, which inspires researchers to develop more adaptive methods, such as ensemble empirical mode decomposition (EEMD), complete ensemble empirical mode decomposition with adaptive noise (CEEMDAN), local mean decomposition (LMD), empirical wavelet transform (EWT), and variational mode decomposition (VMD). As the ship feature express as the multi-modulation phenomenon in the SN signal, each modified method has been introduced to underwater acoustic signal analysis and has achieved great success. In 2017, Li proposed a new ship classification method by using EEMD and IMF energy criterion [15]. In 2019, Li denoised underwater acoustic signals by combining CEEMDAN and least mean square adaptive filter (LMSAF) [16]. In 2022, Tian proposed a multi-layer adaptive separation method based on EEMD for various target detection and experimental results show that multiple targets can be well-estimated even in a complex acoustic environment [17]. Similarly, in 2022, Spinosa proposed a denoising strategy based on EEMD to reduce the background noise in testing of the emergency water landing of aircrafts [18].

As for LMD, EWT, and VMD, they are also concerned in the field of acoustic signal analysis. In 2021, Lu proposed a modified ACELMD method and employed it to extract features from complex underwater acoustic signals. The comparison between ACELMD, ELMD, and LMD shows that ACELMD can effectively reduce the modal aliasing in the decomposition results [19]. In 2021, Li extracted weak features from SN signals by combining EWT and reverse dispersion entropy [20]. In 2017, Li firstly used VMD to decompose underwater acoustic signals and subsequently classified different ships according to multi-scale permutation entropy [21]. Inspired by Li, Hou and Yang proposed VMD-based underwater acoustic signal processing methods in 2021, respectively, and both achieved great success [22,23]. However, these signal decomposition methods suffer from their own intrinsic contradictions. For example, the fixed binary band partition strategy of EWT may divide a mono-component feature into adjacent bands. Meanwhile, the harsh underwater acoustic environment makes it hard to define the mode number and the bandwidth of VMD accurately, which highly influence the accuracy of VMD [24].

Aiming at solving the above-mentioned problems and achieving an adaptive and sparse decomposition, a novel signal decomposition method, named adaptive chirp mode decomposition (ACMD), is developed to extract meaningful segments from a time series [25]. Born from VMD, it can extract characteristic modes adaptively by minimizing their bandwidth, which ensures that each mode contains less noise and meaningless interference. Although it is a recursive method, it can preferentially obtain characteristic modes because of the modulation phenomena of ship features, which reduce the cost of computation. Therefore, ACMD is a more suitable method for SN signal processing than the above-mentioned methods. Firstly, ACMD is employed for rotating machinery fault diagnosis. Chen developed ACMD in 2019 and used it to identify rub-impact fault for rotor-stator systems [26]. In 2020, Yang proposed a new fault detection method for circuit-breakers by combining ACMD and morphological characteristics [27]. In 2021, Srivastava developed a rotor rub model which can detect the onset of rotor rub efficiently whilst revealing the properties of IF of rubbing rotor; ACMD is used to detect the rub and establish the properties of IF during model building [28]. In the same year, Ma exploited a novel fault identification method based on particle swarm optimization (PSO) and ACMD for bearing, and fast SK algorithm is used to get the resonance band signal [29]. Ding proposed a sparsity-assisted ACVMD method, which incorporates a sparsity-assisted IF update scheme that fully exploits the sparse prior of fast-oscillating IF caused by rub-impact fault [30]. Since the SN signal is similar to the vibration signal, ACMD can be introduced into underwater acoustic feature extraction.

The main consideration of this paper focuses on a development of noise reduction and mode selection to perform modified ACMD. Considering that the performance of ACMD is highly influenced by background noise, a modified ACMD method based on the NLmeans is proposed in this paper. Moreover, SK is calculated for mode selection after mode decomposition, which ensures a more reliable feature extraction result.

The organization of the rest of this paper is summarized as follows. Section 2 succinctly reviews ACMD. Section 3 introduces the procedure of the modified method and shows its performance by using simulated signals. In Section 4, two real applications of the proposed method are presented. Finally, the conclusions are drawn in Section 5.

## 2. Theoretical Background

ACMD, which is a tractable version of the variational nonlinear chirp mode decomposition (VNCMD), can analyze multi-modal signals with strongly time-varying modulation characteristics. They both regard the raw signal as a combination of AM-FM signals. Therefore, VNCMD is briefly introduced in the following chapter first and then ACMD with its limitations is illustrated.

### 2.1. A Brief Introduction of VNCMD

A FM-AM signal with *K* sub-signals can be expressed as:

(1) $$s\left(t\right)=\sum_{i=1}^{K}A_{i}\left(t\right)\cos\left(2\pi \int_{0}^{t}f_{i}\left(\tau \right)d\tau +\varphi_{i}\right)$$

where $A_{i}\left(t\right)>0,f_{i}\left(\tau \right)>0$ are instantaneous amplitude (IA) and instantaneous frequency (IF), respectively.$\varphi_{i}$ is the initial phase of the *i*-th component. What should be noticed is that IA and IF vary more slowly than $\varphi_{i}$ does. The purpose of VNCMD is to estimate IA and IF of each sub-signal recursively [31], and its procedure can be shown as follows.

Firstly, Equation (1) can be rewritten by using a demodulation technique:

(2) $$\begin{array}{l} s\left(t\right)=\sum_{i=1}^{K}\left[a_{i}\left(t\right)\cos\left(2\pi \int_{0}^{t}{\tilde{f}}_{i}\left(\tau \right)d\tau \right)+b_{i}\left(t\right)\sin\left(2\pi \int_{0}^{t}{\tilde{f}}_{i}\left(\tau \right)d\tau \right)\right]\\ where\\ \{\begin{array}{c} a_{i}\left(t\right)=A_{i}\left(t\right)\cos\left(2\pi \int_{0}^{t}\left(f_{i}\left(\tau \right)-{\tilde{f}}_{i}\left(\tau \right)\right)d\tau +\varphi_{i}\right)\\ b_{i}\left(t\right)=-A_{i}\left(t\right)\sin\left(2\pi \int_{0}^{t}\left(f_{i}\left(\tau \right)-{\tilde{f}}_{i}\left(\tau \right)\right)d\tau +\varphi_{i}\right) \end{array} \end{array}$$

In Equation (2), the IF of the *i*-th component is $f_{i}\left(\tau \right)-{\tilde{f}}_{i}\left(\tau \right)$ and ideally when $f_{i}\left(\tau \right)={\tilde{f}}_{i}\left(\tau \right)$, the *i*-th component will be purely AM signals, which have the narrowest bandwidths. Therefore, VNCMD tries to estimate all components by minimizing their bandwidth:

(3) $$\begin{array}{l} \underset{\left\{a_{i}\left(t\right),b_{i}\left(t\right),{\tilde{f}}_{i}\left(t\right)\right\}}{\min}\left\{\sum_{i=1}^{K}\left({\left\|a_{i}\prime \prime \left(t\right)\right\|}_{2}^{2}+{\left\|b_{i}\prime \prime \left(t\right)\right\|}_{2}^{2}\right)\right\}\\ s.t.s\left(t\right)=\sum_{i=1}^{K}\left[a_{i}\left(t\right)\cos\left(2\pi \int_{0}^{t}{\tilde{f}}_{i}\left(\tau \right)d\tau \right)+b_{i}\left(t\right)\sin\left(2\pi \int_{0}^{t}{\tilde{f}}_{i}\left(\tau \right)d\tau \right)\right] \end{array}$$

where ${\left\|\cdot \right\|}_{2}^{2}$ is the *L*2 norm and the bandwidth is evaluated using the square of the *L*2 norm of the second-order derivate. Equation (3) can be regarded as a constrained optimization problem and solved by the augmented Lagrange multiplier method, as VMD does.

Generally speaking, the result of VNCMD highly depends on accurate sub-signal number and suitable initial IFs. However, it is scarcely possible to define the number of sub-signals and the initial IFs accurately in real situations because of heavy noise and unknown load. Therefore, a novel decomposition method for real AM-FM signals is desired.

### 2.2. ACMD

Inspired by VNCMD, ACMD can also estimate the IF and IA of the *i*-th mode by minimizing its bandwidth [32]. Motivated by the matching pursuit method, ACMD extracts modes recursively, which means for the *i*-th signal component, the bandwidth optimization problem should be solved as:

(4) $$\begin{array}{l} \underset{a_{i}\left(t\right),b_{i}\left(t\right),{\tilde{f}}_{i}\left(t\right)}{\min}\left\{{\left\|a_{i}\prime \prime \left(t\right)\right\|}_{2}^{2}+{\left\|b_{i}\prime \prime \left(t\right)\right\|}_{2}^{2}+\alpha {\left\|s\left(t\right)-s_{i}\left(t\right)\right\|}_{2}^{2}\right\}\\ with\;s_{i}\left(t\right)=a_{i}\left(t\right)\cos\left(2\pi \int_{0}^{t}{\tilde{f}}_{i}\left(\tau \right)d\tau \right)+b_{i}\left(t\right)\sin\left(2\pi \int_{0}^{t}{\tilde{f}}_{i}\left(\tau \right)d\tau \right) \end{array}$$

where ${\left\|s\left(t\right)-s_{i}\left(t\right)\right\|}_{2}^{2}$ denotes the residue energy after removing the *i*-th estimated component; α is a penalty factor. Like the matching pursuit, ACMD recursively finds the *i*-th component which has the most energy [26]. Considering that SN signals are discrete, Equation (4) can be rewritten as:

(5) $$\underset{u_{i},f_{i}}{\min}\left\{{\left\|\Theta u_{i}\right\|}_{2}^{2}+\alpha {\left\|s-G_{i}u_{i}\right\|}_{2}^{2}\right\}$$

where $\Theta =\left[\begin{array}{cc} \Omega & \\ & \Omega \end{array}\right]$ and Ω is a second-order difference matrix, $u_{i}={\left[a_{i}^{T},b_{i}^{T}\right]}^{T}$ denotes the corresponding demodulated signals which can restructure IA as $A_{i}\left(t\right)=\sqrt{a_{i}^{2}\left(t\right)+b_{i}^{2}\left(t\right)}$, and $G_{i}=\left[C_{i},S_{i}\right]$ with $C_{i}=diag\left[\cos\left(\varphi_{i}\left(t_{0}\right)\right),\ldots ,\cos\left(\varphi_{i}\left(t_{N}\right)\right)\right]$ and $S_{i}=diag\left[\sin\left(\varphi_{i}\left(t_{0}\right)\right),\ldots ,sin\left(\varphi_{i}\left(t_{N}\right)\right)\right]$. Equation (5) means that ACMD is actually a L2-regularized least-squares problem and it can be solved by updating the demodulated signal and the IF function iteratively. The demodulated signal is updated as:

(6) $$u_{i}^{j}=\left[\begin{array}{c} a_{i}^{j}\\ b_{i}^{j} \end{array}\right]=\arg\underset{u_{i}}{\min}\left\{{\left\|\Theta u_{i}\right\|}_{2}^{2}+\alpha {\left\|s-G_{i}u_{i}\right\|}_{2}^{2}\right\}={\left(\frac{1}{\alpha }\Theta^{T}\Theta +{\left(G_{i}^{j}\right)}^{T}G_{i}^{j}\right)}^{-1}{\left(G_{i}^{j}\right)}^{T}s$$

where *j* stands for the time of iterations and the *i*-th component can then be estimated as:

(7) $$s_{i}^{j}=G_{i}^{j}u_{i}^{j}$$

After Equation (7) is gained, we can calculate the frequency increment $\Delta {\tilde{f}}_{i}^{j}$ and subsequently update the IF function:

(8) $$\Delta {\tilde{f}}_{i}^{j}=-\frac{1}{2\pi }\frac{d}{dt}\left(\arctan\left(\frac{b_{i}^{j}\left(t\right)}{a_{i}^{j}\left(t\right)}\right)\right)=\frac{b_{i}^{j}\left(t\right)*{\left(a_{i}^{j}\left(t\right)\right)}^{\prime }-a_{i}^{j}\left(t\right)*{\left(b_{i}^{j}\left(t\right)\right)}^{\prime }}{2\pi \left({\left(a_{i}^{j}\left(t\right)\right)}^{2}+{\left(b_{i}^{j}\left(t\right)\right)}^{2}\right)}$$

(9) $$f_{i}^{j+1}=f_{i}^{j}+{\left(\frac{1}{\beta }\Omega^{T}\Omega +I\right)}^{-1}\Delta {\tilde{f}}_{i}^{j}$$

where *I* is an identity matrix and ${\left(\frac{1}{\beta }\Omega^{T}\Omega +I\right)}^{-1}$ works as a low-pass filter. The updating is executed iteratively until the convergence criterion is satisfied, and then the *i*-th component and the residual signal are gained as:

(10) $$R_{i+1}\left(t\right)=R_{i}\left(t\right)-{\tilde{s}}_{i}\left(t\right)$$

Then, the residual signal $R_{i+1}\left(t\right)$ is regarded as the original signal for the next decomposition until the energy of the residual signal is smaller than a certain threshold. After each IA and IF is obtained, an adaptive TF spectrum is employed to represent the time-varying characteristics of the SN signal:

(11) $$ATFS\left(t,f\right)=\sum_{i=1}^{K}{\tilde{A}}_{i}\left(t\right)\delta \left(f-{\tilde{f}}_{i}\left(t\right)\right)$$

### 2.3. Simulation Validation

**Figure 1:**
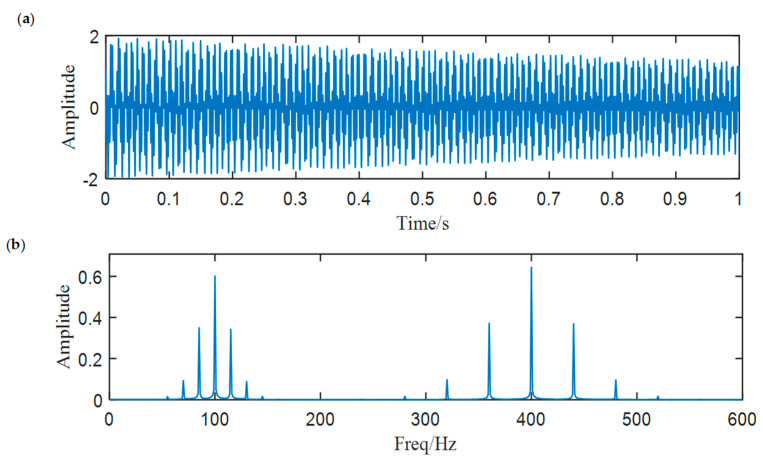
The simulated signal: (**a**) waveform, (**b**) spectrum.

Since ACMD regards signal components as AM-FM signals and extracts modes adaptively, it can extract the SN signal features effectively if the reasonable IFs are set in advance. In order to verify this, we used ACMD to decompose a simulated signal and extract the modulated characteristics mixed in the signal. As shown in Equation (12), the simulated signal is generated by mixing two AM-FM signals, which represent the axial frequency characteristic and the propeller frequency characteristic in the SN signal. The sample rate of the simulated signal we design is 10 kHz and the length of the signal is one second. Figure 1 shows the waveform and spectrum of the simulated signal.

(12) $$\begin{array}{l} x\left(t\right)=x_{1}\left(t\right)+x_{2}\left(t\right)\;\\ x_{1}\left(t\right)=e^{-0.35*t}\cos\left(2\pi *\left(400*t+\frac{1}{2}\pi *\cos\left(2\pi *40t\right)\right)\right)\\ x_{2}\left(t\right)=e^{-0.50*t}\cos\left(2\pi *\left(100*t+\frac{1}{2}\pi *\cos\left(2\pi *15t\right)\right)\right) \end{array}$$

**Figure 2:**
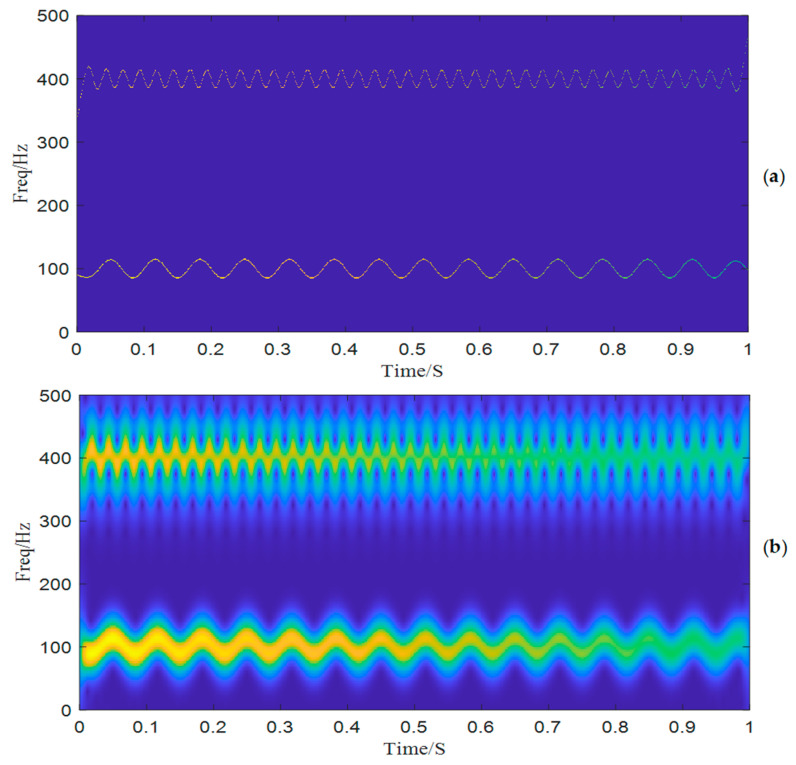
The time-frequency spectrum: (**a**) ACMD, (**b**) STFT.

**Figure 3:**
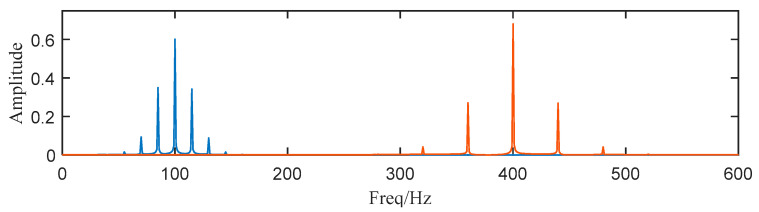
The spectrum of two decomposed modes (Blue: the first sub-signal $x_{1}\left(t\right)$, Red: the second sub-signal $x_{2}\left(t\right)$ ).

Then, we decompose the simulated signal by ACMD, and the TF spectrum with the STFT result are shown in Figure 2a,b. Comparing two figures, we notice that ACMD can distinguish the mixed AM-FM signals more accurately than STFT. The spectrum of the two decomposed modes are also calculated, and the center frequencies 100 Hz and 400 Hz with their modulated frequencies 15 Hz and 40 Hz are clearly shown in Figure 3.

### 2.4. Limitation on Acoustic Feature Extraction

Although ACMD can extract AM-FM components from SN signals effectively, it is still hard to identify weak ship features in practical cases because of heavy background noise. Background noise influence the performance of ACMD from two aspects: firstly, ACMD employs time-frequency ridge detection (TFRD) to gain initial IF function, and the accuracy of TFRD based on STFT depends on a high signal-to-noise ratio (SNR) of the raw signal. Secondly, the constraint of the smoothness of IF functions also makes ACMD sensitive to noise.

**Figure 4:**
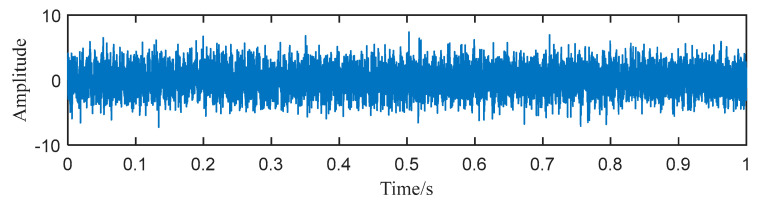
The waveform of the noisy signal.

**Figure 5:**
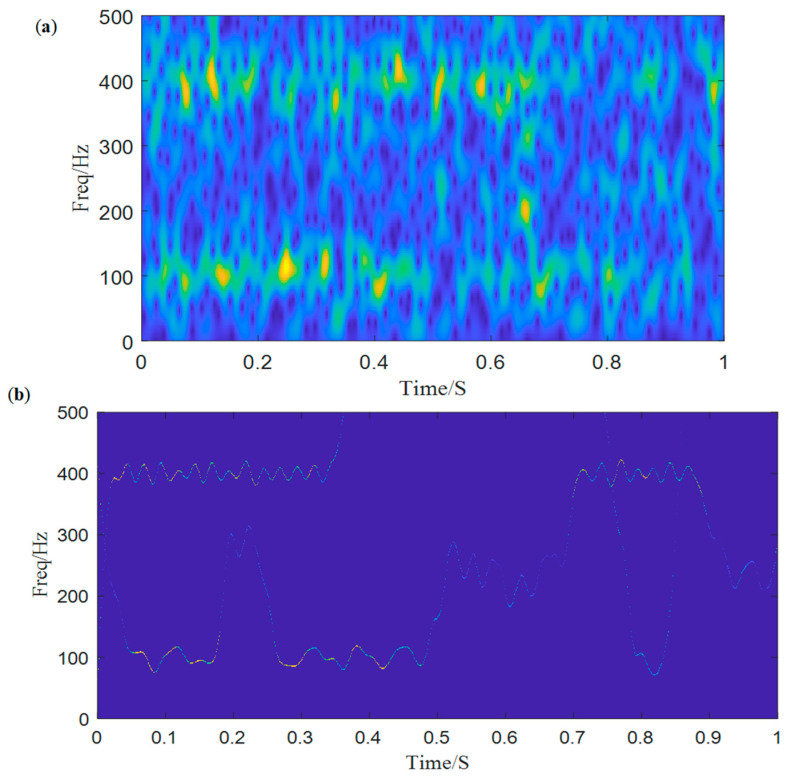
(**a**) The STFT spectrum of the noisy signal, (**b**) the time-frequency spectrum of ACMD.

**Figure 6:**
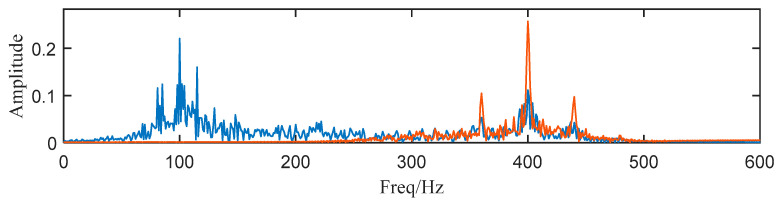
The spectrum of decomposed components (Blue: the first sub-signal $x_{1}\left(t\right)$, Red: the second sub-signal $x_{2}\left(t\right)$ ).

To show the limitation of ACMD, we add white Gaussian noise with the SNR per sample in −5 dB to the simulated signal built in the above section. The new signal is shown in Figure 4, while its STFT spectrum is displayed in Figure 5a. In Figure 5b, it can be noticed that a large number of meaningless components appear, and the ridge lines are indistinct. We still use ACMD to decompose the noisy signal and the result is shown in Figure 6. Comparing Figure 2a and Figure 6, we can notice that the ridge lines representing sub-signal 1 and 2 are totally different. Meanwhile, the spectrum in Figure 6 is also changed because of heavy noise.

Therefore, a plain conclusion can be drawn that some denoising method should be employed before we decompose the SN signal through ACMD. Furthermore, axial frequency characteristics and propeller frequency characteristics should be extracted after signal decomposition, but ACMD trends to decompose the raw signal into several components. Therefore, we need to build an effective criterion to select the mode which contains the most features.

## 3. The Proposed Method

**Figure 7:**
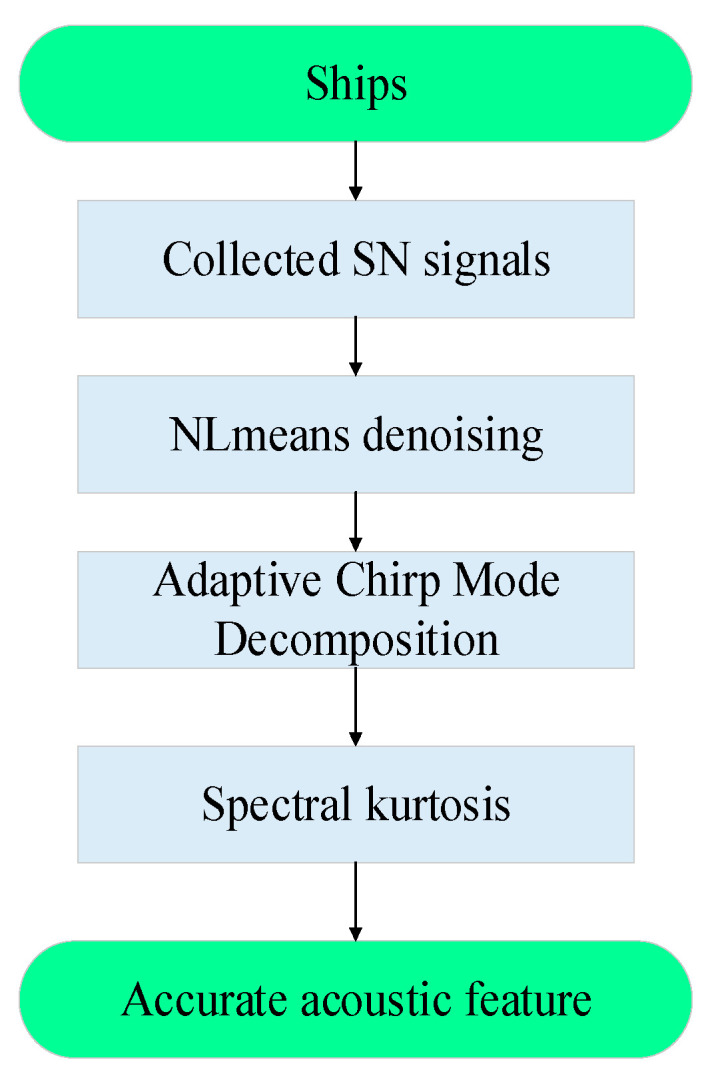
The procedure of the proposed method.

Considering that real SN signals are contaminated by heavy noise and axial frequency characteristics and propeller frequency characteristics are weak, we propose a 3-step underwater acoustic feature extraction method. Firstly, we use NLmeans to denoise the SN signal; secondly, ACMD is employed to decompose the denoised signal into AM-FM components; and finally, SK is applied to select the sub-signal which contains the most ship frequency characteristics, and the chosen sub-signal is demodulated. The procedure is summarized in Figure 7.

### 3.1. The Improved Non-Local Means

There are many denoising algorithms available, such as Wavelet, Morphological Filtering [33], and Matrix Completion [34,35]. To keep the spectral structure of the raw signal, we select NLmeans as the pre-treatment method. NLmeans is proposed by Buades to estimate the true value of each sample point. It can exploit the inherent repeatability of time series to find similar points of a certain sampling point and denoise this point using the average of its similar ones [36]. The original algorithm is explained as follows:

A raw SN signal mixed with an additive noise n(t) can be expressed as:

(13) s(t)=x(t)+n(t)

The estimated value at the time τ is computed as the weighted average of all the signals:

(14) $$\hat{s}\left(\tau \right)=\frac{\sum_{t}\omega \left(\tau ,t\right)s\left(t\right)}{\sum_{t}\omega \left(\tau ,t\right)}$$

And the weighting function can be defined as:

(15) $$\omega \left(\tau ,t\right){=e}^{-\frac{\sum_{\lambda \in \Delta }{\left(s\left(t-\lambda \right)-s\left(\tau -\lambda \right)\right)}^{2}}{B_{\Delta }2h^{2}}}$$

where s(t−λ) means the value of the observed signal at the time (t−λ), Δ represents a local patch of samples surrounding *t* and $B_{\Delta }$ is its total sample. *h* is a smoothing parameter and ${\left\|\right\|}_{a}$ denotes the Euclidean distance based on a Gaussian kernel of a radius *a*. The kernel here can be regarded as a Gaussian window function for filtering.

It has been shown that the NLmeans algorithm can be interpreted as the first iteration of minimizing a general energy functional, and the original exponential form of weighting function is not immutable [37]. However, traditional weighting functions such as LECLERC, HUBER, and LOGISTIC neglect the distance distribution of neighborhoods, so we choose a novel weighting function for NLmeans, which is shown in Equation (16):

(16) $$\omega \left(\tau \right)=\{\begin{array}{cc} 1 & \left\|r\right\|\leq h\\ {\left(1-\frac{{\left(\left\|r\right\|-h\right)}^{2}}{h^{2}}\right)}^{8} & h<\left\|r\right\|\leq 2h\\ 0 & \left\|r\right\|\leq h \end{array}$$

**Figure 8:**
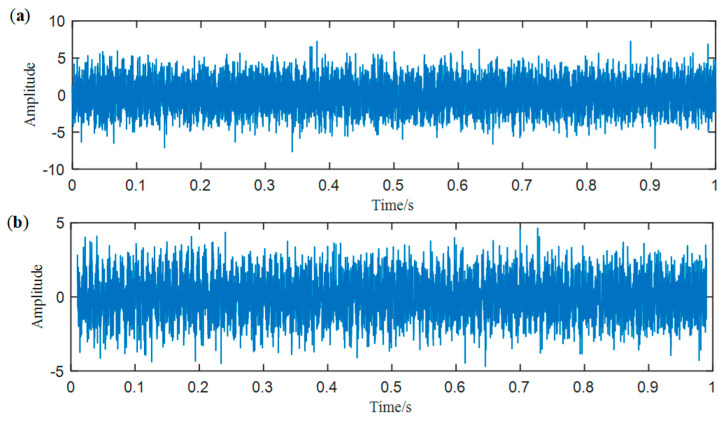
(**a**) The waveform of the noisy signal and (**b**) the waveform of the denoised signal.

In Section 2, we notice that the original ACMD method cannot effectively deal with the noisy signal. Therefore, we use NLmeans with the new weighting function to denoise the simulated signal structured in Section 2. The waveform of the noisy signal and the denoised signal are shown in Figure 8. It can be noticed that simulated impulses can be hardly found in Figure 8a. However, in Figure 8b, clear periodic characteristics can be observed, which shows the performance of NLmeans.

### 3.2. ACMD Algorithm

After denoising, ACMD can be employed to decompose the signal. In a real scenario, we use the correlation coefficient between the raw signal and the reconstructed signal rather than residual energy to judge whether the decomposition loop should be ended. The correlation coefficient is calculated by Equation (17):

(17) $$\rho_{s}=\frac{\sum_{n}\left(s_{n}{}^{\prime }-{\bar{s}}^{\prime }\right)\left(s_{n}-\bar{s}\right)}{\sqrt{\sum_{n}{\left(s_{n}{}^{\prime }-{\bar{s}}^{\prime }\right)}^{2}\sum_{n}{\left(s_{n}-\bar{s}\right)}^{2}}}$$

**Figure 9:**
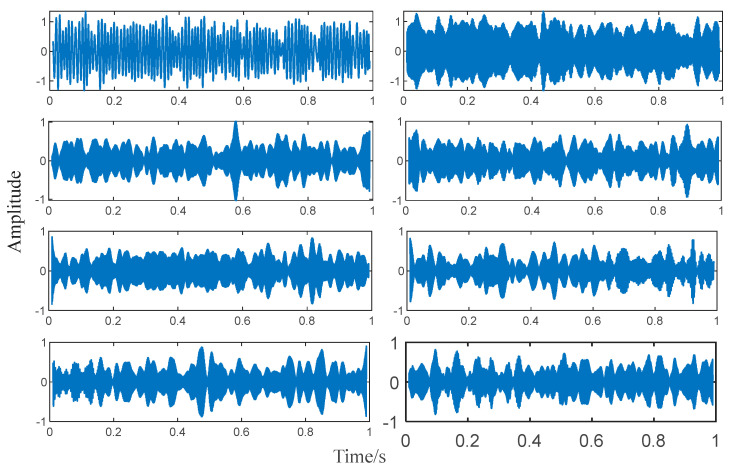
Decomposed sub-signals.

In the simulation, we set the correlation coefficient to be 0.6, which means when the correlation coefficient $\rho_{s}{}^{\prime }\geq 0.6$, the algorithm should be ended. As for the denoised signal, it is decomposed into 8 AM-FM components as shown in Figure 9.

### 3.3. Component Selection Based on Spectral Kurtosis

**Figure 10:**
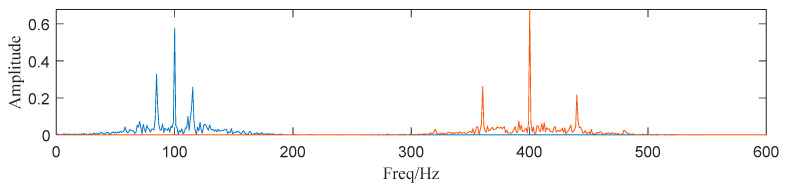
Component 1 and 2 (Blue: the first sub-signal $x_{1}\left(t\right)$, Red: the second sub-signal $x_{2}\left(t\right)$ ).

According to Figure 10, lots of meaningless noise components are generated during the decomposition. Meanwhile, the energy of the noise components may even be greater than components containing ship features when background noise is heavy or when the ship is too far away. Therefore, we introduce SK to select a suitable component for subsequent demodulation analysis. SK [38] can be defined as Equation (18):

(18) $$K_{i}\left(f\right)=\frac{\langle {\left|F\left(n,f\right)\right|}^{4}\rangle }{\langle {\left|F\left(n,f\right)\right|}^{2}\rangle }-2$$

where the operator 〈⋅〉 can be defined as $\langle g\left(n\right)\rangle =\lim_{N\to \infty }N^{-1}\sum_{N}g\left(n\right)$. Usually, SK splits the raw signal into sub-signals according to a 1/2 tree filter-bank and then the SK of each sub-signal is calculated. Now that we have gained all AM-FM components using ACMD, the component with the largest value of SK can be selected as the component containing the most features. The SK of each component is shown in Table 1. Then, we select component 1 and 2 for subsequent analysis. In Figure 10, the structure of the spectrum is highly similar to Figure 1 and Figure 3, which means the proposed method can effectively deal with noisy SN signals.

## 4. Applications

In this section, we use data from the National Park Service and our own hydrophones to prove the effectiveness of the proposed method for SN signals. Moreover, traditional signal decomposition methods, such as EEMD, are also employed for comparison.

### 4.1. Data Collected by National Park Service

Data Introduction and Performance Comparison

**Figure 11:**
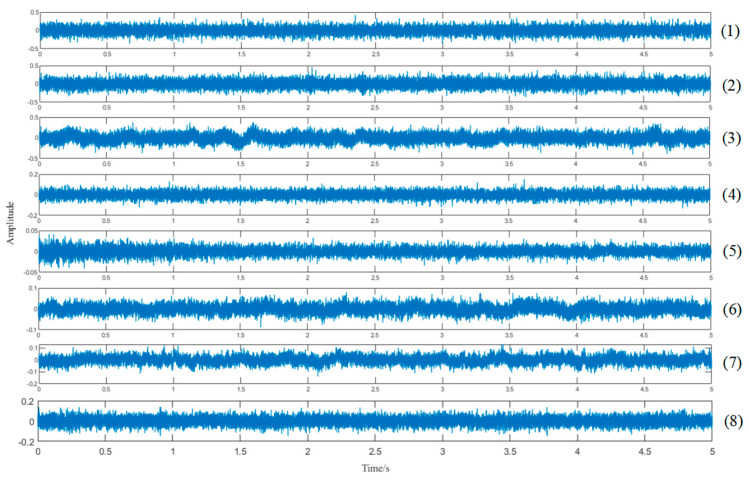
Waveforms of SN signals ((1): a cruise ship, (2): a ferry transporting passengers, (3): a freighter, (4): a passing boat powered by a small diesel engine, (5): a boat with outboard engine, (6): a boat powered by a 60hp outboard engine passes the hydrophone at approximately 10 knots, (7): a boat powered by a 60hp outboard engine passes the hydrophone at approximately 20 knots, (8): a boat with a whining propeller).

The proposed feature extraction method is utilized to analyze real measured ship-radiated noise. The data sets are composed of sound samples radiated from various different ships (cruise ship, ocean liner, motorboat, and ferry), and contain 8 certain SN signals. The wave shapes of 8 SN signals are shown in Figure 11. With respect to each kind of SN signal, we re-sample all SN signals at a sampling frequency of 5 kHz, extract a fragment with 5 s length from each signal for decomposition, and add the same white Gaussian noise to the raw signal.

To show the advantages of the proposed method, we choose a traditional binary-SK method as the comparison object. The comparison result is shown in Table 2. It is obvious that the proposed method has the best ability of feature extraction, with an accuracy of 8/8. Meanwhile, SK has a lower accuracy of 5/8 (the standard is clear spectral lines appearing in the envelop spectra of modes).

**Figure 12:**
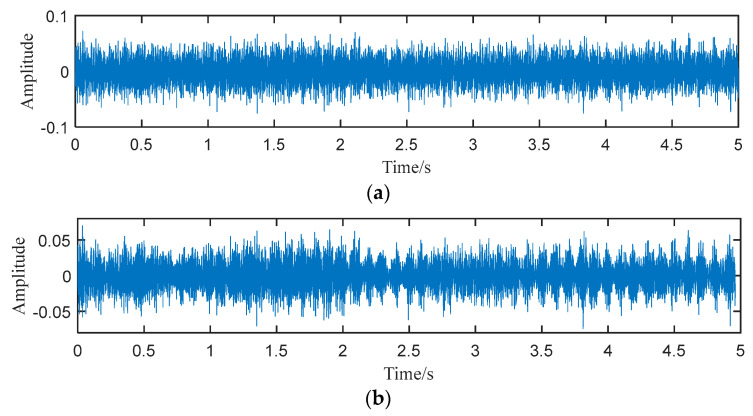
(**a**) Waveform of signal 4, (**b**) waveform of the denoised signal.

**Figure 13:**
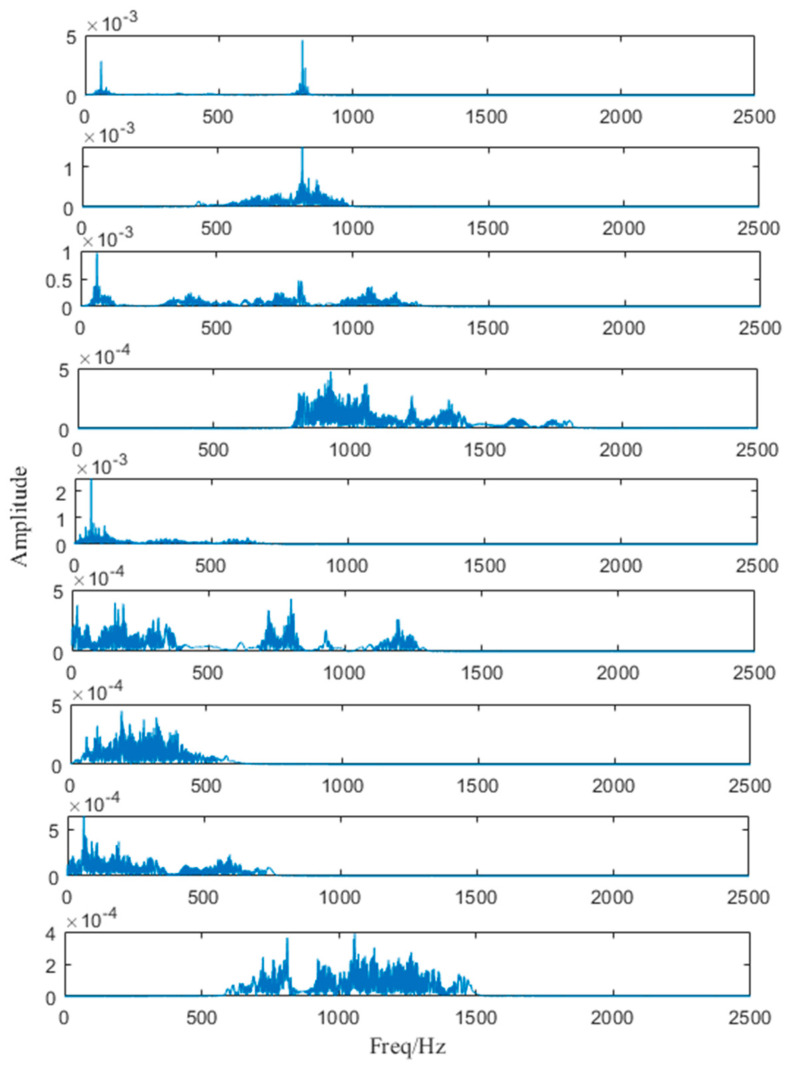
Components divided by ACMD.

Then, we choose a specific SN signal of a small diesel engine (Signal 4) for further comparison. The initial signal is shown in Figure 12a. First, the proposed method is applied for the signal. The raw signal is denoised by NLmeans and periodic pulses are found in the waveform of the denoised signal, as Figure 12b shows. Then, the denoised signal is decomposed by modified ACMD and the result of the decomposition is given in Figure 13. It can be seen that the signal is decomposed into 9 components.

**Figure 14:**
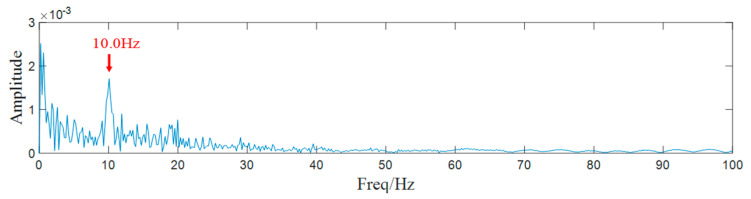
Envelope spectrum of component 1.

Subsequently, the value of SK of each component is calculated and component 1 with the largest SK value is selected for demodulation. The envelope spectrum of component 1 is drawn in Figure 14. In the figure, we can notice that the characteristic frequency 10.0 Hz is clear.

**Figure 15:**
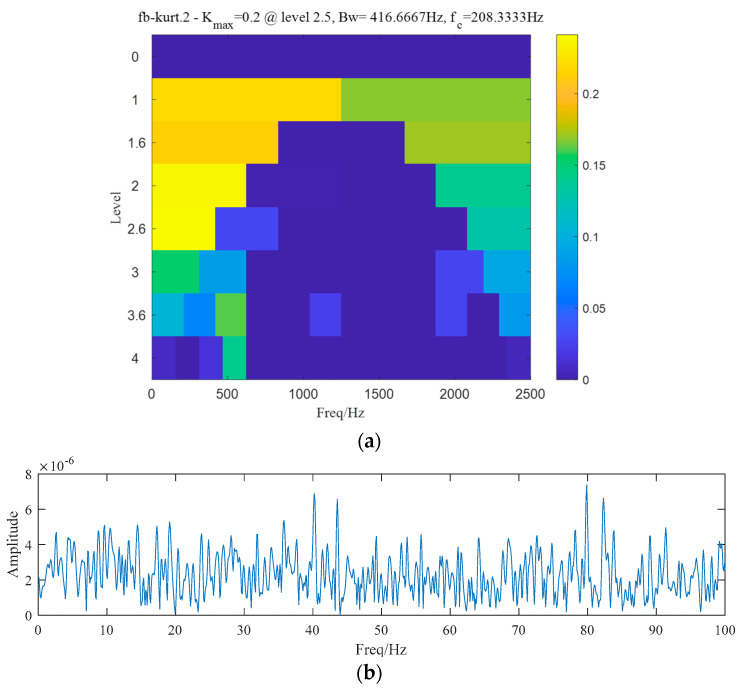
(**a**) Result of a 4-layer SK. (**b**) Envelop spectrum of the band with the largest kurtosis.

A 4-layer SK is also used for comparison and the result is shown in Figure 15a. The sub-signal with the highest SK is located between 0 Hz to 416.66 Hz, but unfortunately no obvious characteristic frequency can be identified from the envelop spectrum in Figure 15b. Therefore, we can conclude that the proposed method shows a better ability of feature extraction for SN signals than SK.

### 4.2. Data Collected by Our Own Hydrophones

**Figure 16:**
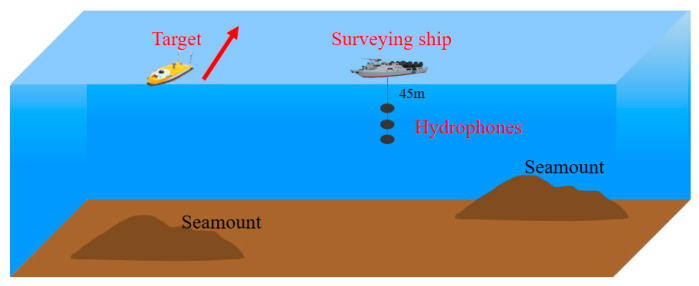
The SN signal exploration and collection experiment.

In 2016, our research group designed and implemented a SN signal exploration and collection experiment in the South China Sea. The surveying ship with three hydrophones and the target ship are shown in Figure 16. The engine of the surveying ship stalls, and the target ship sails with a uniform speed. In the experiment, we collect 61 SN signals with a sampling rate of 20 kHz. Most of the SN signals can be easily analyzed and shafting characteristic frequencies contained are obvious.

**Figure 17:**
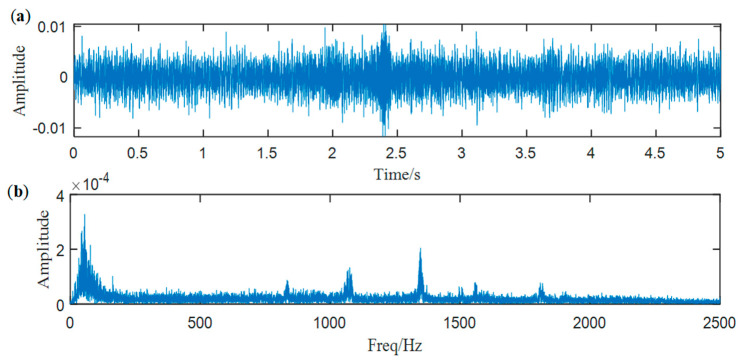
(**a**) Waveform of the SN signal, (**b**) spectrum of the SN signal.

As we gain an effective SN signal processing method, we attempt to process these intractable signals again, and a typical case is illustrated in this section. First, the raw signal and its Fourier spectrum are shown in Figure 17. Because of the complicated underwater acoustic environment and a quite long distance between the surveying ship and the target, the hidden shafting feature cannot be found directly.

**Figure 18:**
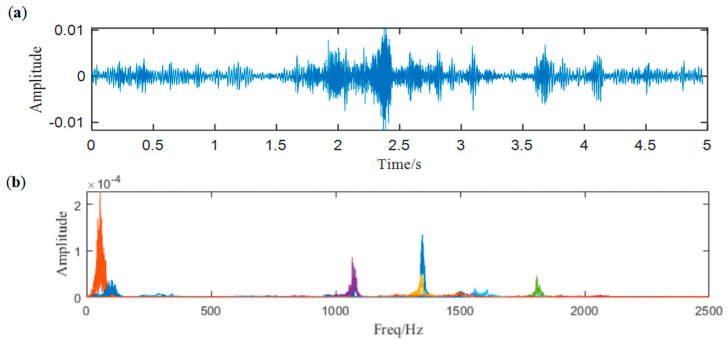
(**a**) Waveform of the denoised signal, (**b**) result of ACMD.

Therefore, the signal is decomposed by the proposed method and the threshold value of correlation coefficient is designed as 0.6. The raw signal is decomposed into 9 components, as Figure 18b shows.

**Figure 19:**
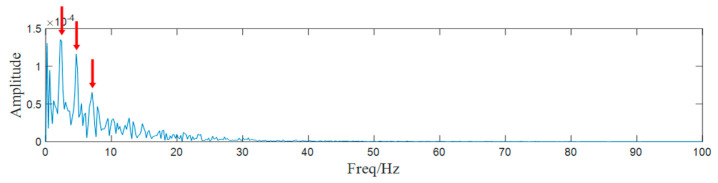
The envelop spectrum of component 3.

Then, the decomposed components are selected according to their SK value and the envelop spectrum of the most suitable component (component 3) is drawn in Figure 19. Clear spectral lines appear in the envelop spectrum and they can be regarded as the shafting characteristic frequency and its harmonics.

**Figure 20:**
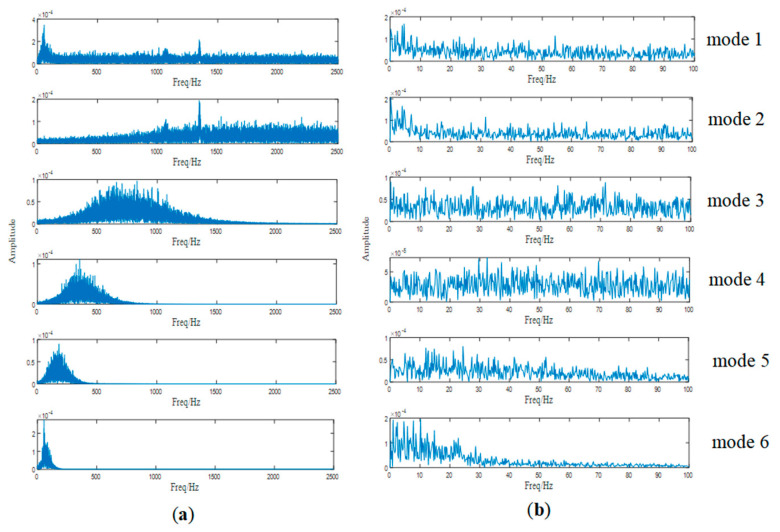
(**a**) The frequency spectra of the first six modes and (**b**) envelop spectra of the first six modes.

As a widely used signal decomposition method, EEMD is also employed to deal with the raw signal for comparison. It divides the signal into 13 IMFs. The spectra of the first six modes are shown in Figure 20a, because higher-order modes are low-frequency narrow-band signals without any modulation information. We demodulate the first six modes and show their envelop spectra in Figure 20b, but it can be noticed that none of the related frequencies appear.

**Figure 21:**
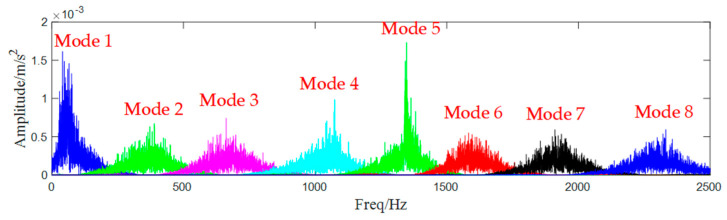
The result of the parameter-optimized VMD method.

Since ACMD is born out of VMD, we also use a parameter-optimized VMD [39] to decompose the same signal for comparison. Coincidently, it also uses SK to optimize its parameters. According to Ref. [39], when the optimal number of modes is 8 and the optimal bandwidth is 1500, the envelope signal kurtosis value takes the maximum of 4.49. Therefore, the raw signal is decomposed by VMD with the optimal mode number 8 and bandwidth 1500; the decomposition result is shown in Figure 21.

**Figure 22:**
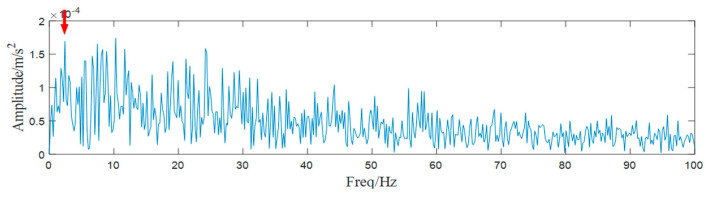
The envelop spectrum of mode 4.

Then, we select mode 4 for demodulation analysis because the kurtosis value of mode 4 is the largest among all 8 modes. Figure 22 shows the envelop spectrum of mode 4, and in Figure 22, we notice that only one spectral line is clear, and the whole spectrum is submerged by expected interference. Therefore, we can draw a conclusion that the proposed method shows a stronger ability for the feature extraction of SN signals.

We analyze the elapsed time of involved methods in both case 1 and 2 and gain the following Table 3 (the algorithms run on a desktop computer with CPU: 11th Gen Intel (R) Core (TM) i7-11700F, Memory: 16 GB, System: Windows 10-19044.2728 and Matlab 2020b).

From Table 1, we can notice that the efficiency of M-ACMD is far lower than EEMD and SK, because M-ACMD is a recursive method and for each mode, it has to update many times to gain the final estimated IF function and estimated IA function. However, SK and EEMD cannot extract the ship features in case 1 and 2; therefore, the elapsed time of M-ACMD is acceptable.

The parameter-optimized VMD costs the longest time because it has to try different combinations of parameters to gain the optimal decomposition result. Compared with the parameter-optimized VMD, M-ACMD can be regarded as an effective method.

## 5. Conclusions and Prospect

### 5.1. Conclusions

SN signal processing and feature extraction play a vital role in ship recognition and classification. In this paper, a modified signal decomposition method based on NLmeans, ACMD, and SK is proposed to deal with SN signals and extract shafting characteristic frequencies.

The modified method has been used in two real cases and compared with other traditional decomposition methods, such as EEMD, SK, and the parameter-optimized VMD. Some benefits of this paper can also be drawn after the above-mentioned work:

1. We introduce ACMD to SN signal extraction and underwater acoustic target identification. Moreover, we build a new correlation-coefficient-based convergence criterion for ACMD instead of the energy of the residual signal.
2. Considering heavy noise of real SN signals, we use NLmeans denoising to improve SNR. However, traditional weighting functions such as LECLERC, HUBER, and LOGISTIC neglect the distance distribution of neighborhoods, so we build a novel weighting function for NLmeans denoising. The modified algorithm has better potential for denoising.
3. We choose SK rather than energy-based criteria as a mode selection criterion because SK is sensitive to ship operation features and insensitive to noise and unexpected interference. The simulation and two cases in Section 4 prove the effectiveness of SK.
4. Both the simulation experiments and real cases show the superiority of the proposed method in weak feature extraction.

### 5.2. Prospect

**Figure 23:**
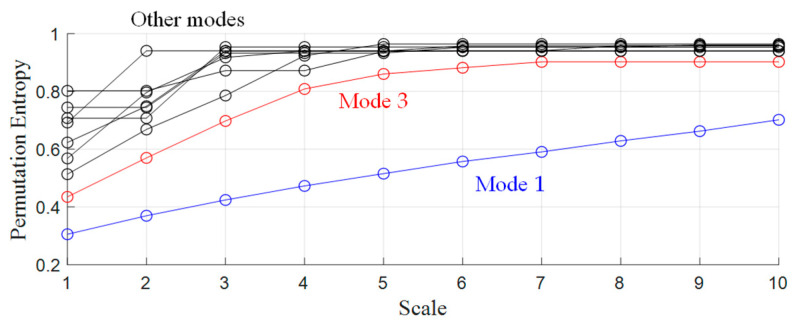
The result of MPE of 9 modes.

Although an effective ship feature extraction method is proposed for taxonomic purposes in this paper, there are still three different ways to improve the potential of underwater acoustic target identification:

1. Sensitive mode selection criteria, such as entropy-based criteria [40,41], can be built and used to assist the feature extraction. Since entropy reflects the degree of disorder of a time series, we can build and calculate the entropy-based index of each mode and choose those modes with the smallest value of the index for demodulation analysis. Take multi-scale permutation entropy (MPE) [42] as a brief example. We calculated the MPE of all 9 modes obtained in case 2 and the result is shown in Figure 23 as follows:

The largest value of PE is 1, meaning that all permutations have an equal probability; the smallest value of PE is 0, indicating that the time series is very regular. In other words, the smaller the value of PE is, the more regular the time series is. It can be noticed that the value of the MPE of mode 1 is the smallest among all modes, but mode 1 is low-frequency signals and always contains strong periodical interference. Therefore, we still choose mode 3 for demodulation analysis and get the same result as that in case 2.

2. ACMD is also a suitable pre-processing method to obtain characteristic modes and can be combined with self-organizing ship identification methods, such as the entropy-based methods. For real SN signals, we can choose a certain mode (such as the first obtained mode by ACMD) and calculate its entropy for ship identification [43]. It is also feasible to calculate entropies of all modes and input them into an artificial neural network for automatic identification.
3. New signal decomposition methods should be proposed. Actually, VMD-based methods, such as VNCMD, FMD and ACMD, can be regarded as constrained optimization problems. Considering that entropy is a significant optimization objective, we can propose new signal decomposition methods by structuring entropy-based objective function and solving the constrained optimization problem.

## Figures and Tables

**Table 1:** SK of each component.

1	2	3	4	5	6	7	8
1360.1	2240.6	7.9	10.1	6.62	16.6	7.8	6.8

**Table 2:** Performance of different methods.

Method	Effective Signals	Samples	Accuracy
M-ACMD	8	8	100%
SK	5	8	62.5%

**Table 3:** The elapsed time of the involved methods(s).

	The Proposed Method	SK	EEMD	Parameter-Optimized VMD [1]
Case1 (25,000 points)	90.22	1.65	/	/
Case2 (25,000 points)	85.47	/	45.77	360.47

## Data Availability

Data is unavailable due to privacy.
